# Tissue-Resident Memory CD8^+^ T Cells: From Phenotype to Function

**DOI:** 10.3389/fimmu.2018.00515

**Published:** 2018-03-26

**Authors:** David J. Topham, Emma C. Reilly

**Affiliations:** ^1^David H. Smith Center for Vaccine Biology and Immunology, University of Rochester, Rochester, NY, United States; ^2^Department of Microbiology and Immunology, University of Rochester, Rochester, NY, United States

**Keywords:** T cells, memory, tissue distribution, integrins, infection, immunity, cellular

## Abstract

Tissue-resident memory CD8^+^ T cells are an important first line of defense from infection in peripheral non-lymphoid tissues, such as the mucosal tissues of the respiratory, digestive, and urogenital tracts. This memory T cell subset is established late during resolution of primary infection of those tissues, has a distinct genetic signature, and is often defined by the cell surface expression of CD69, CD103, CD49a, and CD44 in both mouse and human studies. The stimuli that program or imprint the unique gene expression and cell surface phenotypes on T_RM_ are beginning to be defined, but much work remains to be done. It is not clear, for example, when and where the T_RM_ precursors receive these signals, and there is evidence that supports imprinting in both the lymph node and the peripheral tissue sites. In most studies, expression of CD49a, CD103, and CD69 on T cells in the tissues appears relatively late in the response, suggesting there are precise environmental cues that are not present at the height of the acute response. CD49a and CD103 are not merely biomarkers of T_RM_, they confer substrate specificities for cell adhesion to collagen and E-cadherin, respectively. Yet, little attention has been paid to how expression affects the positioning of T_RM_ in the peripheral tissues. CD103 and CD49a are not mutually exclusive, and not always co-expressed, although whether they can compensate for one another is unknown. In fact, they may define different subsets of T_RM_ in certain tissues. For instance, while CD49a^+^CD8^+^ memory T cells can be found in almost all peripheral tissues, CD103 appears to be more restricted. In this review, we discuss the evidence for how these hallmarks of T_RM_ affect positioning of T cells in peripheral sites, how CD49a and CD103 differ in expression and function, and why they are important for immune protection conferred by T_RM_ in mucosal tissues such as the respiratory tract.

## Tissue-Resident Memory Cells

Tissue-resident memory CD8^+^ T cells (T_RM_) are a distinct memory population that is generated and persists at the site of infection or vaccination (1–3). Upon exposure to the same or similar diseases, T_RM_ cells provide a first line of adaptive cellular defense and are indispensible in lethal challenge models (2, 4). Since CD8^+^ T cells mount responses aimed primarily at more conserved internal epitopes of pathogens, eliciting a T_RM_ response may provide increased protection compared with B-cell targeted vaccines, which some pathogens can escape by mutating antigenic sites (5). While research efforts have started to shed light on some of the requisite signals for maintenance of the T_RM_ population, less is understood in regard to the positioning within the tissue that is required for the development of this subset and responses of T_RM_ cells after rechallenge. Here, we will provide an overview of the gross anatomical locations in which T_RM_ cells form, the known interactions that facilitate their development, and the consequences of reactivation, as well as the voids that remain in our understanding of this critical population. Closing these knowledge gaps will allow us to harness the full potential of T_RM_ and elicit improved protective responses to vaccination.

## Locations

T_RM_ cells are most well known for their roles in barrier sites such as mucosal tissue and skin. While the populations in different tissues display heterogeneity in requirements for migration to the site of infection, the developmental program, maintenance within the tissue, and their surface phenotype, they ultimately perform similar critical functions. In the mucosa, T_RM_ cells have been identified in the intestines, female reproductive tract, salivary glands, tonsils, and lungs (1, 6–10). In addition, T_RM_ or T_RM_-like cells have been described in a number of other non-mucosal sites, including lymphoid and peripheral tissues such as the thymus, spleen, lymph nodes, liver, kidneys, pancreas, heart, skin, and brain (6, 7, 11–14).

In most mucosal and barrier tissues, the T_RM_ population primarily homes to areas of epithelial surfaces, which represent the common site of infections in these tissues. Influenza, for example, infects the airway epithelium and Herpes simplex viruses, the epithelial cells of the skin or cervix (15–18). In other instances, however, such as with cytomegalovirus, the virus infects epithelial cells within an organ (salivary glands) rather than at the barrier surface (19, 20). As one might anticipate based on the name, salivary glands are home to extensive acinar epithelial-rich glandular structures that provide a location for the infection to persist, but also act as an ideal habitat for the formation and maintenance of T_RM_ cells (8). This opens up the possibility that internal glandular structures within other peripheral tissues could be similar targets for T_RM_ and warrants further investigation.

In the lung, CD8^+^ T_RM_ cells preferentially localize to regions rich in collagen IV (ColIV), while CD4^+^ T cells are biased to areas abundant in collagen I (ColI) (21). This coincides with the relative expression of the integrins specific for those collagen types, CD49a and CD49b, respectively (21). In non-mucosal organs, the positioning within the tissue is less well understood. As discussed later in this review, understanding the function of the surface receptors expressed on T_RM_ cells as well as their responses to different chemokine cues may better inform their roles and localization within non-mucosal tissues.

## Transcriptional Regulation of T_RM_

Recent work has focused on identifying the transcriptional regulators of T_RM_; however, these studies suggest that the specific requirements may vary between mice and humans. In mice, CD8^+^ T_RM_ cells from a number of tissues express elevated levels of HOBIT (homolog of BLIMP-1 in T cells) compared with peripheral T cells (22). In conjunction with BLIMP-1, HOBIT promotes maintenance of the T_RM_ population through repression of genes associated with tissue egress. In addition, in mouse CD8^+^ T_RM_, it has been shown that T-bet and Eomes, two T-box transcription factors, needed to be down regulated for the cell to receive signals from TGF-β and upregulate CD103 (23, 24). However, a low level of T-bet expression is required to maintain expression of the IL-15 receptor β-chain (24, 25). Thus, these two T-box transcription factors control receptiveness to TGF-β and IL-15 signals, which are necessary for proper T_RM_ formation and function (9, 23–25).

In humans, a different set of transcription factors appear to be critical for T_RM_ development and maintenance. Similar to mice, Eomes and T-bet were not expressed in the T_RM_ subset (26). However, the link between HOBIT expression and T_RM_ cells is less evident. HOBIT is expressed in both circulating and resident CD8^+^ T cell populations in humans, and when identified, associates more with cells lacking markers of residence. Reinforcing this notion, a more recent study evaluated gene profiles of CD69^+^CD8^+^ T cells derived from various human tissues and found low to absent levels of HOBIT (27). Instead, these cells expressed NOTCH-1 and HIF-1α, where NOTCH-1 regulated T_RM_ metabolism, which was suggested as its major function (26). In other cases, organ-specific transcriptional regulators were identified. In the lung, RUNX3, BATF, AHR, AP-1, RBPJ, and NF-κB were detected in the T_RM_ subset (26). Many of these regulate T cell effector functions and homing receptors. In the small intestine and vaginal mucosa, a requirement for mTOR was found through inhibition with rapamycin treatment (28). This defect was attributed to an inability to migrate to the site and respond to antigen, and less with a failure to be maintained within the tissue.

Despite some insight into the transcriptional control of T_RM_ cells, few of these transcription factors identified appear to be “master regulators” of T_RM_ differentiation as they are expressed in other CD8^+^ effector or memory subsets. This suggests that T_RM_ differentiation and maintenance is likely controlled by complex combinations of several transcription factors, and the requirements may differ between mice and humans.

## Functions of Surface Proteins that Regulate T_RM_ Localization

It has become clear that one of the only ways to concretely define a cell as “resident” is through the use of parabiotic mice, which allow for equilibration of all circulating cells, but not cells of residence (29, 30). However, the majority of studies in both mice and humans have used expression of a panel of T_RM_ identifiers: CD103 (integrins αE paired with β7), sphingosine-1-phosphate receptor 1 (S1P_1_) antagonist CD69, collagen-binding CD49a (integrin α1 paired with β1), and hyaluronic acid (HUA) binding CD44 as surrogates. It is worth noting that not all populations of T_RM_ cells display all of these markers, suggesting there may be some nuance in T_RM_ subsets. The functional ramifications of expressing any of these molecules likely fit with the requirements for the appropriate positioning of the cells and long-term survival in the tissue.

## CD69

The lectin CD69 is an antagonist of S1P_1_ and limits egress by blocking responsiveness to sphingosine-1-phosphate gradients (31–33). It complexes with S1P_1_ on the cell surface, which leads to its internalization and degradation (34). CD69 is initially upregulated on recently activated effector cells that have seen their cognate antigen, perhaps to limit egress from the lymph node, but constitutive expression is only observed on resident cells (35, 36). Downregulation of Krüppel-like factor 2 on these cells ultimately allows for the expression of CD69 within peripheral tissues (37). Interestingly, CD69 expression is not limited to CD8^+^ T cells, and its presence on other immune subsets including natural killer cells and certain peripheral dendritic cells (DCs) plays a similar functional role of maintaining them within the organ (38, 39). While CD69 is expressed on the majority of T_RM_ subsets, absence of this lectin on CD8^+^ T cells only limits the size of the population and does not result in complete ablation (12). This suggests that CD69 is not an absolute requirement, and while its expression may be advantageous, it is not mandatory. T cells, including T_RM_, are dynamic in their movement in different tissue settings, and in peripheral tissues, multiple retention factors are important for maintaining the resident population. As previously insinuated, populations of T_RM_ cells exist within the salivary glands and female reproductive tract that are CD69 negative. This is interesting given the requirement for downregulation of the S1P_1_ to establish the T_RM_ population in the salivary glands; however, it further suggests that other mechanisms of organ retention are at play (37, 40). So far, little is understood in regard to whether CD69 plays any role in how the cells are positioned within the tissue. In fact, it is quite possible that its primary and only role is limiting their exit from the organ to return to the blood and lymphatics.

## CD103

Integrin αE (CD103) pairs with integrin β7 and is upregulated upon exposure to the active form of TGF-β (41, 42). The most well-known function of CD103 is as a receptor for E-cadherin, an adherans junctional protein interlocking epithelial cells (41). CD103:E-cadherin interactions can act as a tether, which may aid in positioning, retention, and the shape of cells within the epithelium (43, 44). Skin T_RM_ cells lacking CD103 are fewer in number and exhibit increased motility compared with their wild-type counterparts, corroborating this role *in vivo* (12, 45). Similarly, CD103 deficiency results in lower numbers of CD8^+^ T_RM_ cells in the lung after influenza infection (46) and a decrease in intestinal CD8^+^ T cells responding to oral *Listeria* infection due to a defect in initial accumulation (47). Since epithelial cells are the targets for a number of mucosal viral infections, adherence and localization of T_RM_ cells to the epithelium positions them to act as the first line of defense in subsequent exposures. In this regard, CD103 also facilitates the generation of a T_RM_ population at tumor sites such as in the case of melanoma (48). In fact, T_RM_ production by mucosal vaccination leads to inhibition of tumor growth in a preclinical model of head and neck cancer, which was substantiated through parabiotic experiments in mice (49).

While physical retention through ligand binding is the most obvious role for CD103, engagement of CD103 may have a number of other functional ramifications outside of adhesion. While the effects of CD103 binding have been primarily studied in tumor models, the identified features of this integrin are likely widespread throughout various disease states. CD103^+^ tumor-infiltrating CD8^+^ T cells are more capable of killing tumor cells (50). This is likely attributed to the fact that CD103^+^ T cells form more stable synapses with target cells than their CD103-negative counterparts (51). Engagement of CD103 also positions cytolytic granules to organize in a polarized fashion, and the addition of signaling through the TCR results in lytic granule exocytosis (52, 53). Although these functions of CD103 are redundant in the presence of CD11a (LFA-1), T_RM_ cells, especially in the airways of the lungs, display low levels of LFA-1 (54). In fact, LFA-1 levels have been used to determine the age of the T_RM_ cells in the airway, functioning as a clock and decreasing over time (3). One hypothesis is that airway T_RM_ cells are not cytolytic because the synapse stability is affected by this defect. However, CD103 expression on T_RM_ may compensate for low LFA-1 levels and promote effective cytolytic responses to secondary infections.

Moreover, engagement of CD103 may also function to directly position the cells within a given tissue. As an example, it has been shown in the tumor microenvironment that binding of CD103 results in the upregulation of the chemokine receptor CCR5 (55). This suggests that the integrin/chemokine axis could greatly affect the downstream consequences of migratory cues received by a cell and looking at each pathway discretely may limit the overall understanding of the response. In the lung, CCR5 is critical for CD8^+^ T cells to reach the airways (56). Therefore, it would not be unreasonable to hypothesize that CD103 deficiency may alter the localization of the CD8^+^ T cells and delay clearance of the infection. On the flip side, binding of CCL25 through chemokine receptor CCR9 contributes to expression of CD103 on CD8^+^ T cells in the intestine (57). While it is relevant that chemokine signals other than TGF-β may contribute to the upregulation of this integrin on the surface, due to limited expression of CCR9 on CD8^+^ T cells in other organs, this may be a gut-specific mechanism.

In addition to E-cadherin expression on epithelial cells, flow cytometric analysis has demonstrated that the protein is expressed on the surface of specific immune populations such as DCs and, in some instances, T_RM_ cells (58–60). In the salivary glands and gut, E-cadherin is detected on virus-specific CD8^+^ T cells, a phenomenon specific to mucosal compartments, as their lymphoid counterparts in the spleen do not display this phenotype (60). While it is unclear whether the CD8^+^ T cells are producing E-cadherin naturally or acquiring the surface phenotype through a process such as trogocytosis, there are likely to be functional consequences of non-epithelial cells adorned with E-cadherin (61, 62). This may allow for the formation of more stable synapses, in this case between T cells and their APC, or potential cell:cell communication *via* engagement of ligand on other T cells. Surface E-cadherin on the T_RM_ cell could alternatively lead to homotypic interactions (63) in the absence of CD103 expression; however, the downstream functional consequences of this interaction are unclear.

The brain and other epithelium-sparse tissues pose a conundrum in regard to understanding the role that CD103 plays on T_RM_ cells. In the brain, T_RM_ cells are localized to borders of different anatomical regions and in some cases in proximity to vasculature (64). Despite this, expression of CD103 does not appear to corroborate with positioning. However, as just posed, one possibility is that CD103 solely interacts with E-cadherin expressing immune cells in regions devoid of epithelial surfaces and potentially localized similarly to the profile of the T_RM_ cells. Another possibility is that CD103 has other unexplored ligands at play. Although only identified in an *in vitro* system, it has been suggested that CD103 can bind to microvascular endothelial cells derived from the intestine (65). While this did not hold true with endothelial cells derived from the dermis, it has yet to be investigated in the brain and other non-mucosal organs and could better identify a functional role for CD103 in these locations.

Overall, one of the caveats when studying the roles of CD103 *in vivo* is that deletion of the gene results in a lack of DCs expressing this marker. CD103^+^ DCs are critical for the development of T_RM_ cells in the lung during influenza infection and are one of the APC population that drains to the mediastinal lymph node early during infection to present antigen (30, 53). One of the approaches to circumvent this issue is to transfer CD103 knockout transgenic cells into WT mice. This is the approach employed to establish its requirement in the skin, lungs, and gut as previously described (12, 46). However, none of these studies addressed whether CD103 signaling may be required during early stages of development and could ultimately alter the population prior to becoming established at a peripheral site. To fully examine the role of CD103 in the development and persistence of CD8^+^ T cells, it would be necessary to develop an inducible knockout specific to CD8^+^ T cells, so that the integrin could be eliminated at specific time points postinfection.

## CD49a

The role of CD49a on memory T cells in peripheral tissues was discovered in 2004 (2). At that time, the term T_RM_ had not yet been coined, although it has since been associated with this subset of memory T cells (2, 9, 12, 66, 67). CD49a, or integrin α1, pairs with CD29 (integrin β1) to form the heterodimer VLA-1. VLA-1 is a collagen-binding integrin, with preference for the non-fibrillar form, ColIV (68, 69), although it can also bind to ColI, the fibrillar form present in the interstitium of almost all tissues (21). Early studies showed that VLA-1 was not only critical for adherence to ColIV but also migration of the cells along the collagen (68–70). The idea that two of the predominant integrins expressed on T_RM_ cells have opposing functions when interacting with the tissue is quite remarkable, and yet both CD49a and CD103 appear to contribute to the development and/or survival of this population.

While CD49a does not have a direct role in attaching to epithelial cells, its ligand ColIV is located in the lamina densa layer of the basement membrane of mucosal epithelium and is the surface to which the epithelial cells are attached (71). The motility features of this interaction could allow for migration along the basement membrane to access additional regions of the epithelium or may be essential for traversing the collagen to reach infected cells. In either scenario, this interaction is believed to be critical for persistence of the resident population as demonstrated in both the lungs and the intestines (2, 72). While some have assumed that CD49a acts to retain T cells at the epithelium, this has not been unequivocally demonstrated experimentally. As mentioned, it could be necessary to migrate within those sites. CD49a regulation of retention and motility is not mutually exclusive as the ability to stay in the tissue and perform surveillance are the functional hallmarks of T_RM_.

In addition to fostering close proximity to the target cells, engagement of CD49a has a pro-survival role. In conjunction with signaling through the TNF receptor II, binding to CD49a works in a synergistic fashion to protect the cells from undergoing apoptosis (73). Blocking CD49a with antibodies results in a diminution of T_RM_ in mucosal sites (2). Similarly, genetic deletion of CD49a results in the resident population becoming limited (2, 72). Protection requires the presence of a sufficient number of T_RM_ cells, and mice deficient in CD49a become susceptible to secondary heterosubtyptic infections at an earlier time point after primary infection (2–4).

While the requirement for CD49a expression on T_RM_ cells became clear through mouse focused studies, T_RM_ from human subjects share many attributes. In the lung, T_RM_ expressing CXCR6, CD49a, CD103, and CD69 are known to be the major memory population (27, 74). In the skin of healthy individuals, CD8^+^ T_RM_ cells expressing CD49a identify a population of cells that produce IFNγ (9). Unexpectedly, in addition to IFNγ, when stimulated with IL-15, they produce large amounts of perforin and granzyme B (9), perhaps offering a potential mechanism for their reactivation as effectors in secondary encounters. On the other hand, CD49a-negative CD8^+^ T cells in the skin instead produced IL-17 and were associated with psoriatic lesions, indicating that CD49a expression defines different functional subsets. Whether CD49a was required to establish or maintain these cells was not investigated. In mice, CD49a^+^ CD4^+^ memory T cells in the lung provide rapid effector and innate like functions, probably related to more efficient recruitment of other effector cells. This effector function bias based on CD49a expression has not yet been reported for T_RM_ in other tissues.

## CD44

A fourth marker of T_RM_ cells is CD44. While CD44 alone does not distinguish T_RM_ cells from other CD8^+^ T cell populations, its continued expression suggests that similar to other surface receptors, CD44 may have a functional role in T_RM_ biology (75). CD44 is a C-lectin containing glycoprotein, which is expressed on various cell types, including leukocytes and epithelial cells (76). The most well-studied function of CD44 is as a receptor for HUA, a component of the extracellular matrix, and a substance made by vascular endothelial cells and an array of immune cells (77, 78). HUA expression in peripheral tissues is upregulated during inflammation and increases hydration of the tissue (79). On CD8^+^ T cells, CD44 is a classical marker of previous activation, expressed on newly generated effector cells as well as resting memory cells (80). Unlike other T cell markers, which are either on or off, CD44 is expressed in a gradient depending on whether a cell is naive, an effector, or a memory cell (81, 82). Of note, CD44 exists in alternatively spliced isoforms, with various posttranslational modifications, which result in differential affinity for ligand (83, 84). However, in mice, only the invariant form of CD44 has been identified on T cells, with only suggestions that alternative forms are transiently expressed during periods of immunological challenge (85).

In regard to accessing peripheral tissues during an immune challenge, CD44 can bind HUA expressed on vascular endothelial cells and facilitate transmigration (86). In addition, CD44 can interact with CD49d through their intracellular domains, which may enhance this process. However, complete deletion of CD44 does not limit the accumulation of CD8^+^ T cells at the site of initial infection, suggesting that this role is redundant when the cells can utilize other selectins expressed on the cell surface. Once within the tissue, CD44 expressed on DCs improves TCR synapse stability, possibly through binding to CD8^+^ T cell production of HUA (87, 88). Expression of HUA on other immune cell subsets could also aid in cell:cell communication.

Most relevant to migration of CD8^+^ T cells is that CD44 has been shown to play a critical role in maintaining cell structure through adherence to ECM (89). In addition to binding HUA, CD44 can interact with other matrix proteins such as fibronectin, laminin, and collagen. Loss of CD44 during *in vitro* experiments demonstrated that cells no longer display stable polarization with extension of a uropod (89). This defect resulted in a decreased migratory capacity within peripheral tissue, which affects initial access of the tissue. CD44 ligand binding led to recruitment of a number of signaling molecules, which transmitted signals to the cytoskeleton to stabilize the cellular morphology (89).

In the T_RM_ population, CD44 is maintained at high levels, suggesting that it may be important for the T_RM_ population; however, the specific function has yet to be elucidated (75). One possibility is that CD44 could be acting as a general receptor for ECM, maintaining cell shape and polarity as the cells interact with collagen and epithelial cells (89). However, this has yet to be directly tested. An essential role for CD44 in survival of Th1 CD4^+^ T cells has been demonstrated; however, this function was not identified on CD8^+^ T cells (90). In fact, so far the majority of functional requirements for CD44 are enhanced on Th1 CD4^+^ T cells and do not translate to the CD8^+^ T cell compartment (90). However, its role within the T_RM_ subset has not been comprehensively interrogated. Understanding the biology behind the contribution of CD44 to the localization and/or maintenance of the CD8^+^ T_RM_ population could shed light on a new pathway of regulation and warrants further investigation.

## Roles of Cytokines and Chemokines in Positioning the Cells within the Tissue

The roles of chemokines in proper positioning within lymphoid organs have been extensively studied (91). However, their contribution within peripheral tissues is less well understood. In the lung, absence of CCR5 on T cells prevents CD8^+^ T cells from effectively reaching the airway and clearing influenza infection (56). Alternatively, CXCL12 packaged in neutrophil trails facilitates efficient migration of CXCR4^+^CD8^+^ T cells to the site of influenza infection (92). However, the chemokine cues that are required for the acute response may or may not translate to the T_RM_ population and long-term protection. Depletion of neutrophils delayed CD8^+^ T cell infiltration into the infected lung but did not affect the development or protective capacity of T_RM_ (93). By contrast, it is clear in the intestine that expression of CCR9 and consequent binding to CCL25 is essential for migrating T cell subsets to localize to the epithelium and for the development of memory (57).

In the skin, treatment with pertussis toxin decreases the velocity of CD8^+^ T_RM_ cells and alters their morphology, resulting in a rounded phenotype (45). This suggests that chemokines likely play a role in the formation of dendritic spines on the T cells and interactions with the tissue. Keeping with this, the development of the T_RM_ population in the skin greatly benefits from the presence of CXCR6, which is one of the “core” markers of bona fide T_RM_ from many tissues (27, 45). The chemokine that this receptor binds is CXCL16, which has been shown to position innate lymphoid cells and natural killer T cells in tissues and is released by DCs during viral infection (94, 95). Although a diminished population still develops in the absence of CXCR6, the great reduction suggests that it increases either entry into tissue or proper positioning for survival. Alternatively, in the tonsils constitutive expression of IL-15 in the T cell zones and the subepithelium retained virus-specific CD8^+^ T cells within these two locations (96). Interestingly, in this organ, CD103 was only expressed on the T cells in close proximity to the epithelial cells and not in the extra-follicular region.

In many of these cases, signalizing through a cytokine or chemokine receptor may have other implications that indicate that the molecule itself is not directly necessary, but rather the downstream effect such as integrin activation or expression. For example, as previously addressed, interplay between TGFβ and CCL25 are associated with an upregulation of CD103 (57). Engagement of CD103 in turn leads to upregulation of CCR5 (55). CCR5 and CX3CR1 on CD8^+^ T cells in the lung are important for positioning of the cells in proximity to highly inflamed sites and the presence of antigens (56). These consequences suggest that there are complicated signaling pathways and loops at play that have yet to be fully elucidated.

In addition, the presence of various cytokines may provide sufficient inflammatory signals to allow cells access to locations not previously available. Inflammation of the vascular endothelium and changes to the barrier epithelium can occur either through cytokines or early immune cytolytic responses to infection (97, 98). Both of these events could dynamically alter the tissue landscape and allow T_RM_ cells to gain access to previously obscured regions and persist. To fully understand these changes, it is likely that *in vivo* imaging systems will need to be developed to examine alterations in the ECM over time.

## Functions of T_RM_ Cells

While this was touched on briefly earlier in the review, the ultimate role that T_RM_ cells play in protecting a host is not fully understood. One possibility is that the cells produce antiviral cytokines and control downstream immune responses to eliminate infected cells. Alternatively, T_RM_ cells could directly target the cells. In mice, T_RM_ cells in the lungs make large quantities of IFN-γ in response to antigen-specific rechallenge, and blocking of this antiviral cytokine is detrimental to survival (99). In this instance, cells do not produce granzymes or other markers of cytotoxicity. T_RM_ in the female reproductive tract display a decrease in motility after reexposure suggesting that the cells are interacting with antigen-bearing DCs; however, the direct antiviral outcome was not examined in this context (100). CD4^+^CD49a^+^ T cells in the lung activate within hours of secondary challenge with influenza and secrete an array of chemokines that attract other immune effectors (101). In line with this, CD4^+^-resident memory cells in the lungs are also sufficient for promoting airway hyperresponsiveness in a house dust mite model of asthma (102); however, this response can be partially attributed to T_RM_ activation of local DC subsets (103).

Reactivation of human T_RM_ cells alternatively not only leads to IFNγ production but also degranulation of cytolytic granules, including granzyme B and perforins (9, 74). It is conceivable that the limited responses observed in the mouse models are due to the methods employed to evaluate the cellular responses. It is possible that T_RM_ cells are cytolytic *in vivo* with the proper stimulus. Other signals may be necessary for a similar response *in vitro*. For example, T_RM_ cells may require signals from CD103 as well as through the TCR for degranulation of lytic granules. To fully understand the responses, it is necessary to examine these cells *in vivo*. To achieve this, granzyme B reporter mice and other protein reporter mice are the ideal tools (104, 105). As an alternative approach, transcriptional reporters for cytokine production or calcium reporters to indicate productive interactions with APCs may suffice (106–108). Utilizing these tools in conjunction with reporter pathogens, all the cues and responses necessary for protection of the host can be elucidated (109).

## Other Open Questions

With the current methods employed for examining T_RM_ cells, researchers are likely underrepresenting the populations as demonstrated by Steinert et al. (40). Using histology and/or other imaging methods will better reveal the totality of the T_RM_ cell presence and ultimately their response. Although T_RM_ cells are identified through expression of the discussed surface receptors, it is still not clear how the combination of markers alters the response of a given cell. Not all cells that express these markers at the resolution of an infection persist in the organ, suggesting that it is a combination of expression profiles, localization within a tissue, and perhaps proximity to APCs, which may present persistent antigen.

While CD49a, CD103, CD69, and combinations of chemokine receptors are used to define T_RM_, the precise positioning of the T cells in mucosal and glandular tissues remains to be directly examined, including demonstration that CD103 actually binds to E-cadherin at epithelial sites. Besides positioning, how these markers regulate cell motility versus retention in different tissues is not well defined. Intravital microscopy may be one approach to answer some of these questions.

It is also still not fully understood what population of cells lead to the T_RM_ subset. In the lung, evidence suggests that T_EM_ may be maintained within the parenchyma and refeed the transitory airway subset (30). Other T_RM_ populations may also be self-renewing, similar to other populations of resident immune cells which seed organs earlier during development (11, 110). In fact, recent evidence in the female reproductive tract suggests that T_RM_ cells divide during antigen-specific challenge and contribute to the secondary T_RM_ population more than circulating memory cells (100).

Given the common features of T_RM_ subsets in different tissues begs the question of whether there is a common developmental program, as no one specific pathway has been identified. How that program is triggered remains unclear, although TGF-β is required for CD49a and CD103 expression (23). Identifying key features, such as route of delivery, will be critical for optimally generating T_RM_. Previous studies indicate that memory generated through direct infection or exposure of the tissue is distinct from cells that result from systemic priming (47). Ideally, production of T_RM_ could be an effective approach to problems like a universal flu vaccine; however, the field still struggles with eliciting an effective response in a vaccination setting.

## Conclusion

**Figure 1:**
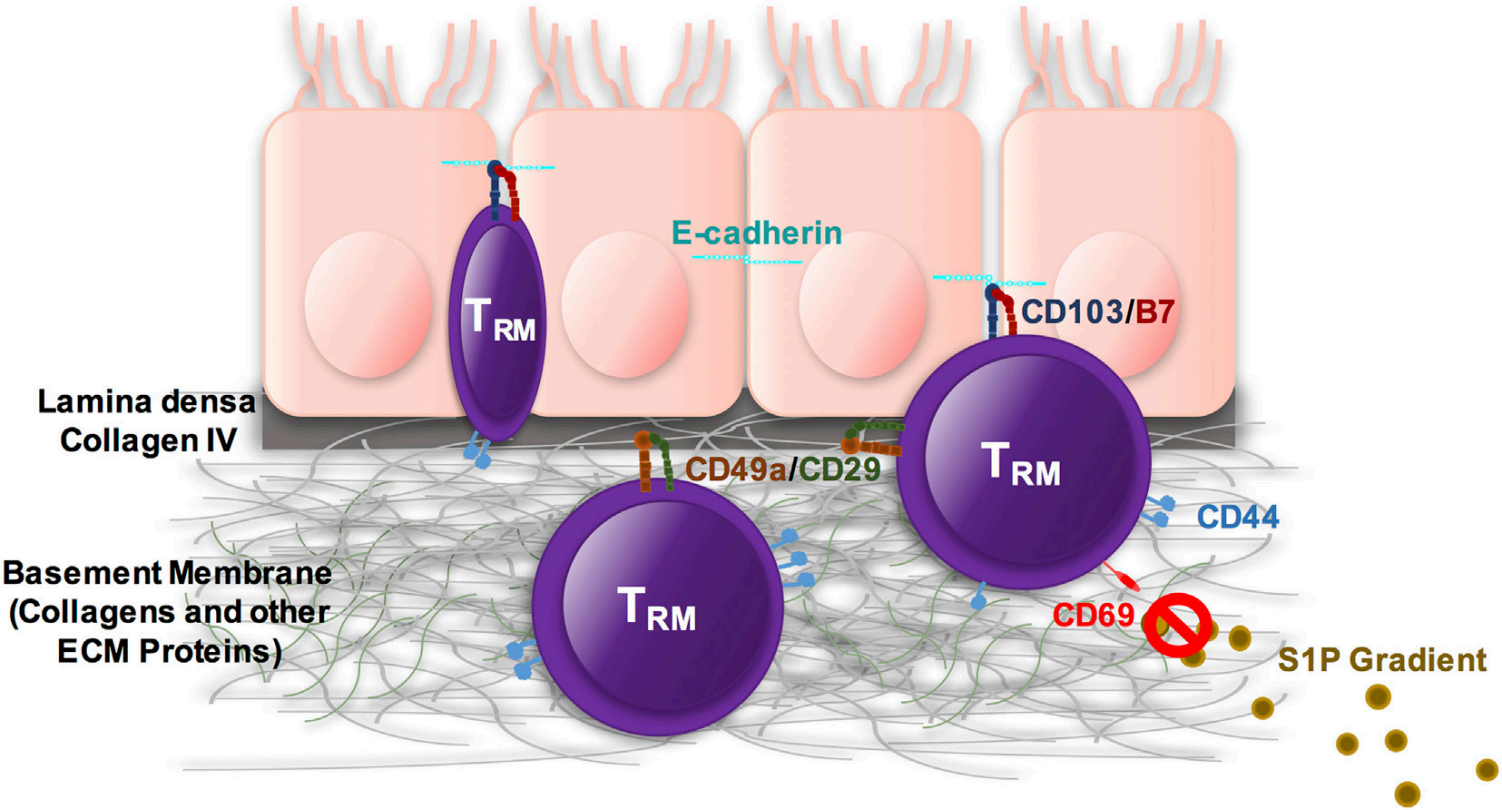
TRM cells are heterogenous both within tissues and also between sites. They express some combination of CD49a, CD103, CD44, and CD69, which cooperate to position the cells and maintain them within the site of initial infection. CD103 can interact with E-cadherin within epithelial surfaces. CD49a interacts with collagens with a preference for collagen IV in the lamina densa underlying the epithelium. CD44 maintains cell shape and integrity and can interact with a number of different tissue components including hyaluronic acid as well as fibronectin and other ECM proteins. CD69 antagonizes S1P_1_, essentially blocking any response to S1P gradients.

T_RM_ cells are important for protection from secondary encounters with various pathogens. They can protect and preserve the integrity of barrier surfaces such as skin, gut, and respiratory tissues. Transcriptional regulation is different in mouse and human systems, although a set of “core” markers have been identified, each having a distinct role in establishing and maintaining T_RM_. It is clear from these studies that T_RM_ cells are highly regulated, poised to respond, yet suppressed by molecules including PD-1 and CD101 to prevent aberrant activation (27). Although the specific cues that result in loss of that suppression have not been identified, it is likely a combination of signals that may include type 1 interferons, which are one of the earliest innate factors made during infections and TCR engagement. In terms of positioning, there is evidence that CD49a interacts with ColIV, and CD103 adheres to E-cadherin, both retaining the cells near the epithelial surface (Figure 1). CD69 and CD44 do not seem to have the same level of necessity, and they are not included in the list of core markers identified in humans; however, they both likely contribute to persistence through limiting egress and maintenance of cytoskeletal structure. To resolve many of these questions future work will need to be done to elicit a full understanding of how T_RM_ cells function to provide pathogen surveillance and protection.

## Author Contributions

All authors listed have made a substantial, direct, and intellectual contribution to the work and approved it for publication.

## Conflict of Interest Statement

The authors declare that the research was conducted in the absence of any commercial or financial relationships that could be construed as a potential conflict of interest.
